# Intrahepatic Cholangiocarcinoma With Rare Metastasis Locations in a Younger Patient: A Case Report

**DOI:** 10.7759/cureus.99787

**Published:** 2025-12-21

**Authors:** Inês P Carvalho, Adalmira Gomes, Bárbara Pena, Inês Bonito, Célia Carmo

**Affiliations:** 1 Internal Medicine, Centro Hospitalar Barreiro-Montijo, Barreiro, PRT; 2 Anatomical Pathology, Centro Hospitalar Barreiro-Montijo, Barreiro, PRT

**Keywords:** advanced stage disease, clivus metastasis, headache, intrahepatic cholangiocarcinoma, persistent diplopia, younger patients

## Abstract

Intrahepatic cholangiocarcinoma is a highly aggressive tumor, usually diagnosed in late middle age and often at an advanced stage. In this clinical case, we report the case of a 41-year-old woman presenting with diplopia, blurred vision, and headache for three days. Imaging investigations showed metastases in the liver, bone, lung, clivus, and rare locations for this type of cancer, such as the spleen and a soft tissue lesion with pre-protuberance expression. The biopsy of the liver lesions confirmed intrahepatic cholangiocarcinoma as the primary tumor. Awareness should be raised regarding the possible atypical symptoms of this cancer and the potential increase in incidence among younger age groups, which may be associated with worse outcomes.

## Introduction

Intrahepatic cholangiocarcinoma is an aggressive malignant tumor that originates from the epithelial cells lining the intrahepatic bile ducts and is the second most common primary liver cancer after hepatocellular carcinoma [1-2]. It primarily affects older adults, with the typical age at diagnosis in the 60s [3-5], and is rare in individuals under 50 [6-7]. Several risk factors are associated with this tumor, including chronic liver diseases, biliary diseases, and diabetes; however, many cases occur without identifiable risk factors [2, 8-9]. The liver and lymph nodes are the most common initial sites of spread, often indicating advanced disease [10-12]. Here, we describe the case of a 41-year-old woman recently diagnosed with advanced intrahepatic cholangiocarcinoma, presenting with diplopia and headache as the main symptoms. We find it important to report this case not only to highlight the atypical manifestations and extent of metastasis but also to raise awareness that the incidence of this tumor is increasing [1], including among younger age groups.

## Case presentation

A 41-year-old Caucasian woman with a known history of common variable immunodeficiency and related recurrent respiratory infections, asthma, and hypothyroidism seeks medical evaluation, complaining of diplopia, blurred vision, and an occipital headache that extends to the frontal area. These symptoms appeared suddenly and have been present for three days, with no other associated symptoms like fever, vomiting, nausea, or altered mental status.

The patient denied cigarette smoking, alcohol use, recent travel, or having a history of autoimmune disease, chronic hepatitis, or biliary tract disease. There was also no family history of cancer.

On physical examination, the patient was unable to perform abduction of the right eye, suggesting paralysis of the lateral rectus muscle and possible involvement of cranial nerve VI. No other neurologic symptoms or signs were present. Examination of other organ systems revealed no additional findings except for a painless enlarged liver (hepatomegaly) on abdominal exam, which was not accompanied by jaundice, pruritus, ascites, palmar erythema, or spider hemangiomas. No other organomegaly or adenomegaly was palpable.

A cranioencephalic computed tomography (CT) scan was performed, revealing a lytic lesion of the clivus on the right side and a soft tissue lesion component with pre-protuberance expression. The CT imaging was supplemented with magnetic resonance imaging (MRI), which confirmed the presence of two lesions on the clivus - one median and another paramedian - and identified an additional lesion on the left side of the frontal bone.

**Figure 1:**
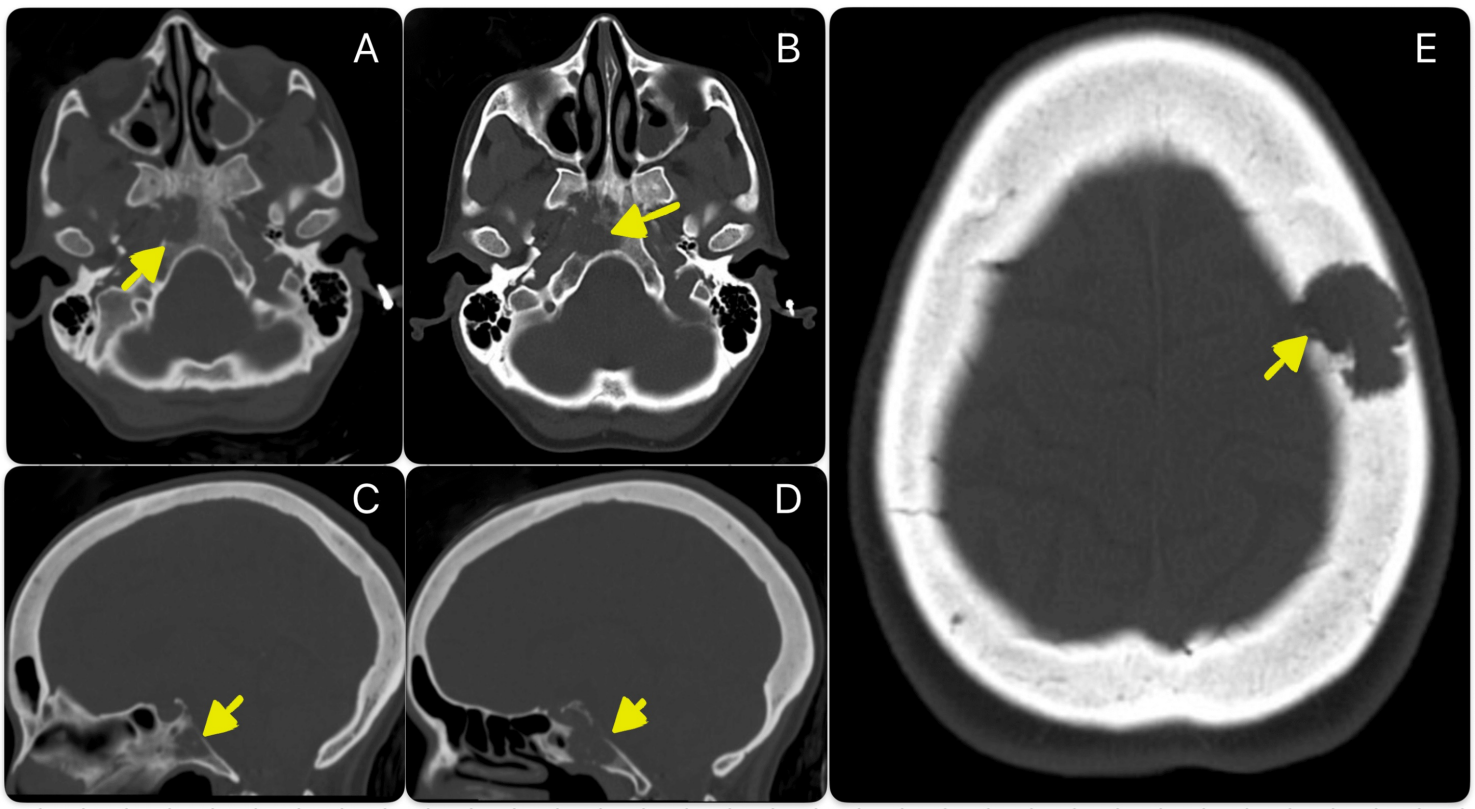
Cranioencephalic computed tomography scan CT scan performed at admission showing a lytic lesion of the clivus (transverse section of cranial CT scan - A, B; sagittal section of cranial CT scan - C, D) and an additional lesion on the left side of the cranial vault (transverse section of cranial CT scan - E).

**Figure 2:**
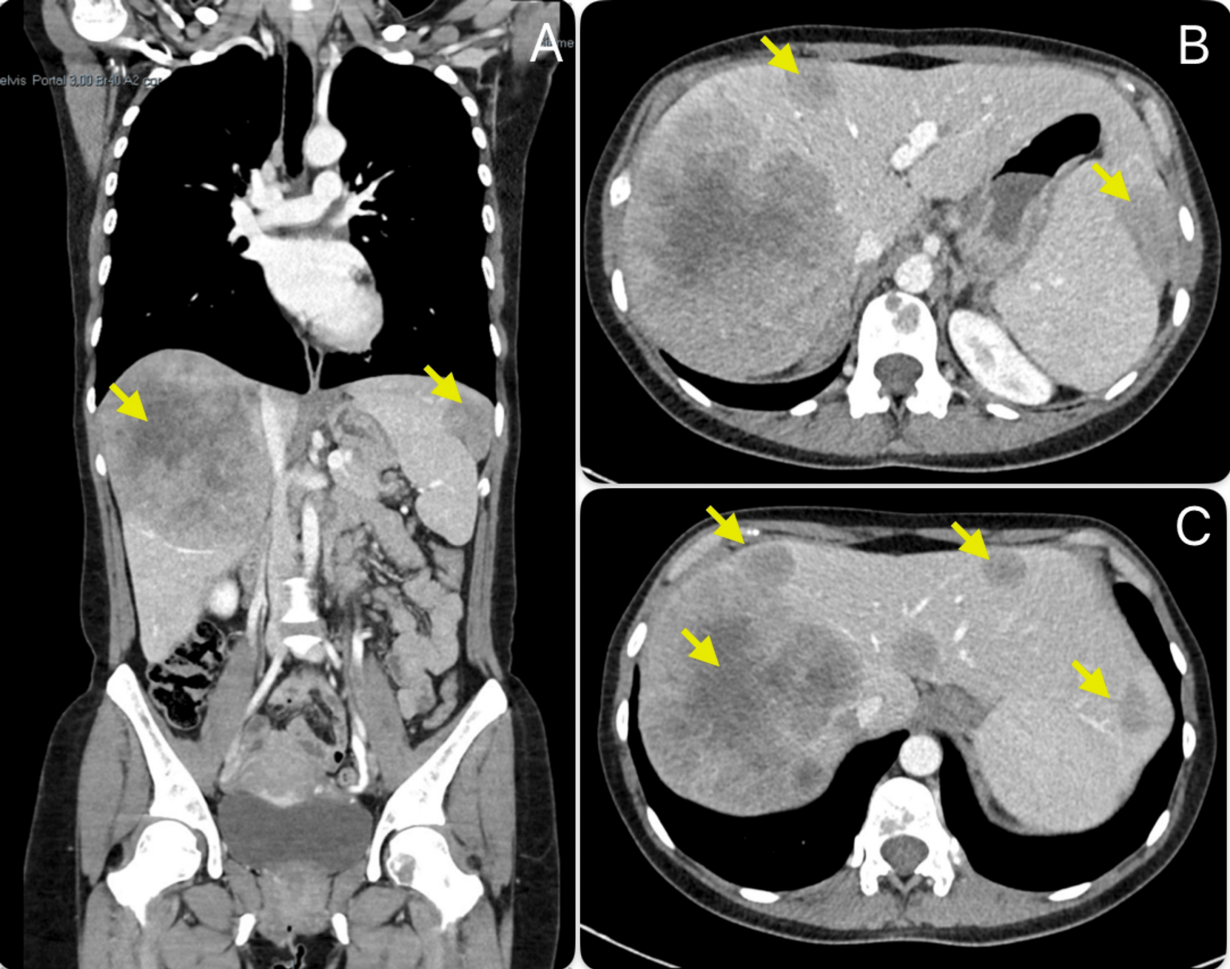
Full body computed tomography scan CT scan showing several hepatic metastases and a splenic metastasis (coronal section of abdominal CT scan - A; transverse section of abdominal CT scan - B, C).

**Figure 3:**
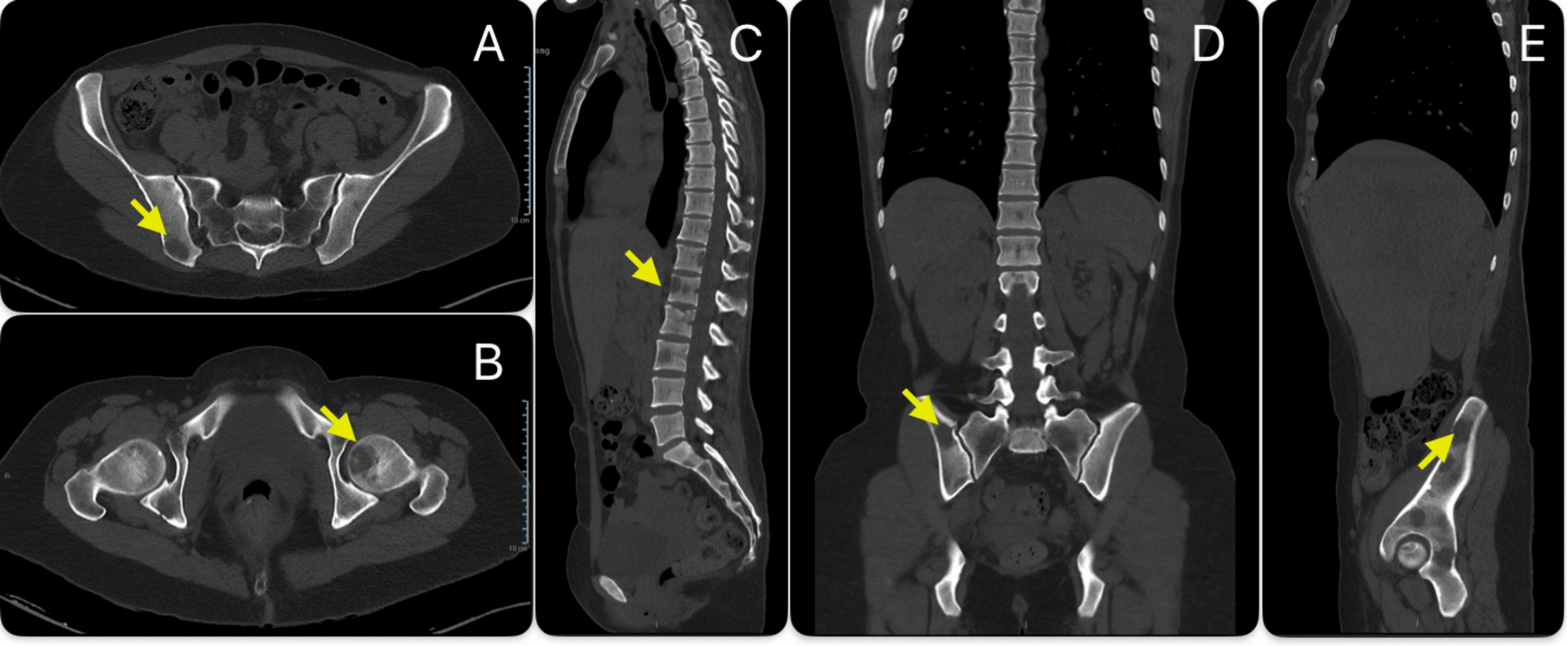
Vertebral spine and pelvic computed tomography scan Numerous lytic lesions were observed in the vertebral spine and pelvis (transverse section of pelvic CT scan – A, B; sagittal section of vertebral and pelvic CT scan – C, E; coronal section of vertebral and pelvic CT scan – D).

Assuming the metastatic origin of the lesions, a full-body CT scan performed later revealed multiple adenopathies (in the mediastinum, pulmonary hila, and left lumbo-aortic chain), several hepatic metastases (causing significant hepatomegaly), and a single metastasis in the lung and spleen. Numerous lytic lesions were also observed in the vertebral spine and pelvis. The findings on CT are shown in Figures 1-3.

None of these findings was accompanied by changes in blood tests, particularly hepatic transaminases and bilirubin, which remained consistently normal. Serologic tests for hepatitis and HIV also yielded negative results. There was an elevation in tumor markers, specifically cancer antigen (CA) 125 (472.5 U/mL, normal range <35), CA 15.3 (41.9 U/mL, normal range <31), carcinoembryonic antigen (CEA) (10.7 ng/mL, normal range <5), and cytokeratin fragment (CYFRA) 21-1 (79.73 ng/mL, normal range <2.08).

**Figure 4:**
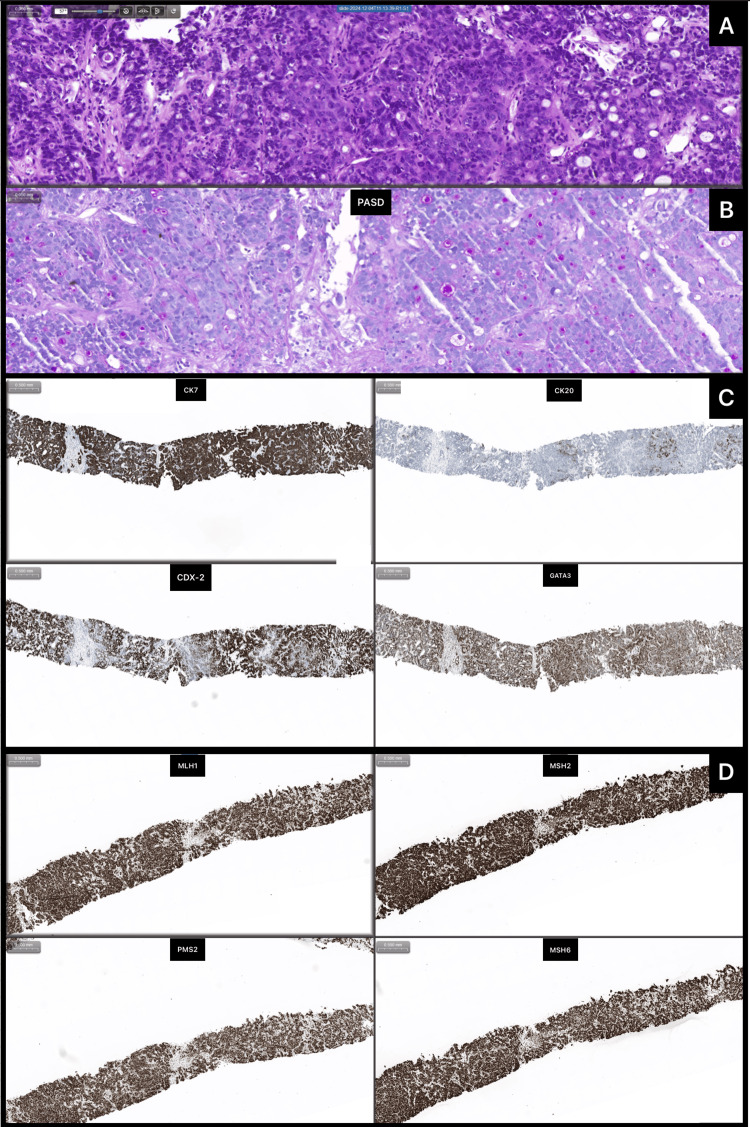
Anatomopathological characterization of hepatic metastasis fragments Fragments obtained with fine-needle biopsy contain an adenocarcinoma with tubular or cord-like patterns, as demonstrated with haematoxylin-eosin coloration (A); in the first pattern, there are glands with variable-sized lumen and, in the latter, a slit-like lumen also occurs along with a desmoplastic reaction with hyalinized fibrous stroma. The carcinoma cells are small to medium-sized, cuboidal, with small nuclei containing fine chromatin and scant cytoplasm. With the aid of histochemical studies (B), mucin-secreting glands are evidenced (PAS-d positive). Immunochemically (C), the neoplastic cells are positive for CK7, CDX2, and GATA-3, with scarce CK20; the mismatch repair proteins are intact, preserving their normal function (D). These histopathological aspects are diagnostic of an intrahepatic cholangiocarcinoma of large duct type.

To identify the primary tumor, a biopsy of one of the hepatic lesions was performed. The anatomopathological diagnosis showed liver infiltration by adenocarcinoma with a cord-like and microglandular pattern with rosette-like outlines. It was positive for PAS-Diastase stain and immunohistochemically positive for CK7, CK20, CDX2, and GATA 3, while negative for CD56, synaptophisin, chromogranin A, glipican, HAS, inibin, mammaglobin, PAX8, RE, RP, TTF-1, and WT1. These findings are consistent with intrahepatic cholangiocarcinoma, G2. The anatomopathological findings are shown in Figure 4.

The patient started dexamethasone and opioid therapy for symptom management and was referred to an oncology appointment. However, treatment was not possible because the woman developed severe signs and symptoms of disseminated intravascular coagulation, which led to her death.

## Discussion

Intrahepatic cholangiocarcinoma, a tumor mainly seen in older adults, is linked to high mortality due to its silent onset, late diagnosis, and resistance to treatment [1, 8, 13], and its incidence is rising.

This disease’s aggressiveness is clearly demonstrated in the case presented here, with the patient exhibiting extensive metastasis, including in locations not usually linked to this tumor, such as the spleen, clivus (which is rare even in other tumors [14]), and a soft tissue lesion component with pre-protuberance expression. Additionally, there are very few disease manifestations, including one that is uncommon for this tumor type (diplopia).

Furthermore, the patient’s young age is another important aspect to highlight, as diagnoses at this age represent a small percentage of cases and the patient did not exhibit risk factors usually associated with this type of tumor that could explain its occurrence at a younger age (major risk factors include chronic liver diseases like hepatitis B or C, cirrhosis, biliary diseases like primary sclerosing cholangitis, hepatolithiasis, liver fluke infection, diabetes or obesity [9, 14]). The fact that the patient had common variable immunodeficiency might have contributed to the development of this cancer at this age, as this disease is linked with a higher risk of malignancy and liver problems (likely due to chronic inflammation, immune dysregulation, and recurrent infections) [15, 16]. However, the patient never showed signs of pre-existing liver disease, and the connection between common variable immunodeficiency and intrahepatic cholangiocarcinoma is limited in the current literature.

## Conclusions

This case concerns a patient with complaints of paralysis of the lateral rectus muscle, diplopia, and blurred vision due to metastasis of an advanced and aggressive intrahepatic cholangiocarcinoma. The condition appeared at a younger age and with metastasis in locations rarely described in other reported cases. Given these peculiarities, the report of this case aims to raise awareness not only of the increasing incidence of aggressive tumors in younger patients (who may have few or atypical signs and symptoms) and the necessity of new tools to diagnose these tumors quickly and improve the poor prognosis associated with them, but also of the importance of biopsy in cases where the primary tumor is not easily identified.
